# Supplementary material for: Selectively exciting quasi-normal modes in open disordered systems
Source: Nat Commun. 2018 Nov 9;9:4714. doi: 10.1038/s41467-018-07180-3 (PMC6226460; doi:10.1038/s41467-018-07180-3)
Supplement: Supplementary file 1 — Supplementary Information [file 41467_2018_7180_MOESM1_ESM.pdf]

Supplemental Information for 'Selectively exciting quasi-normal modes in open disordered systems'

Davy *et al.*

Supplemental Information for 'Selectively exciting quasi-normal modes in open disordered systems'

Matthieu Davy[1] and Azriel Z. Genack[2]

[1]Institut d'Electronique et de Télécommunications de Rennes, University of Rennes 1, Rennes 35042, France

[2]Department of Physics, Queens College and Graduate Center of the City University of New York, Flushing, New York 11367, USA

Supplementary Note 1 - Decomposition into modal components.

We confirm in recursive Green's function simulations that the modal transmission matrices (MTMs) are of unit rank. The MTM is obtained from spectra of the TM for two samples with different values of the conductance g; $g = 0.1$ ($N = 16$) and $g = 1.1$ ($N = 33$). Excellent agreement is found in both cases in Supplementary Figure 1. The contribution of each mode to $T(\omega)$, $T_n(\omega)$, is also shown. We also compare the second and first eigenvalues of $t_n^\dagger t_n$ to the transmission eigenvalues, which are eigenvalues of $t(\omega)^\dagger\, t(\omega)$. The ratio of the two first eigenvalues of the MTM is found to be typically 10^{-8} in strongly localized samples and 10^{-6} in diffusive samples with greater modal overlap.

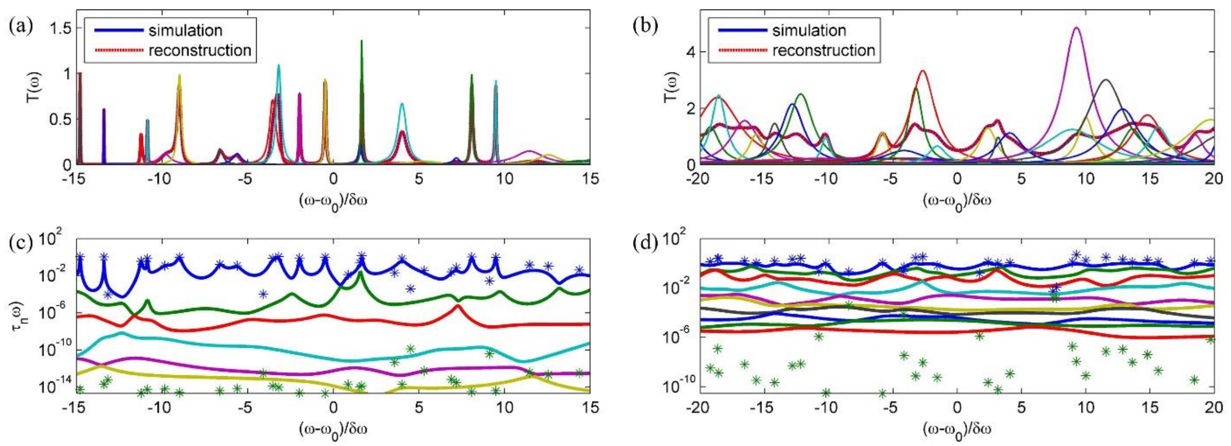

Supplementary Figure 1| Decomposition of simulated spectra into MTMs. (a) Transmittance (blue curve) and its reconstruction found using HI (dashed red curve) for a sample with $g = 0.1$ and $N = 16$. (c) The corresponding transmission eigenvalues and the two first eigenvalues of the MTM are shown on a semilog scale. (b,d) Same as (a,c) but for a sample with $g = 1.1$ and $N = 33$.

Further confirmation that the MTM is of rank unity is found in measurements in a weakly disordered system with moderate spectral overlap. The TM is measured between 8.8-9.25 GHz, which is a frequency range in which the antennas are weakly coupled to the sample. The cavity contains 150 randomly placed Teflon disks of index of refraction, $n = 1.44$. The coupling strength of the antennas is $\tilde{T}_a \sim 0.15$. The leakage from the cavity is therefore small and resonances are narrow. $\delta = 0.6$ in this case and modes extend throughout the sample even though the degree of overlap is weak. The reconstruction of $T(\omega)$ is presented in Supplementary Figure 2(a). We find the ratio of the two first eigenvalues of the MTM to be $\sim 10^{-4}$.

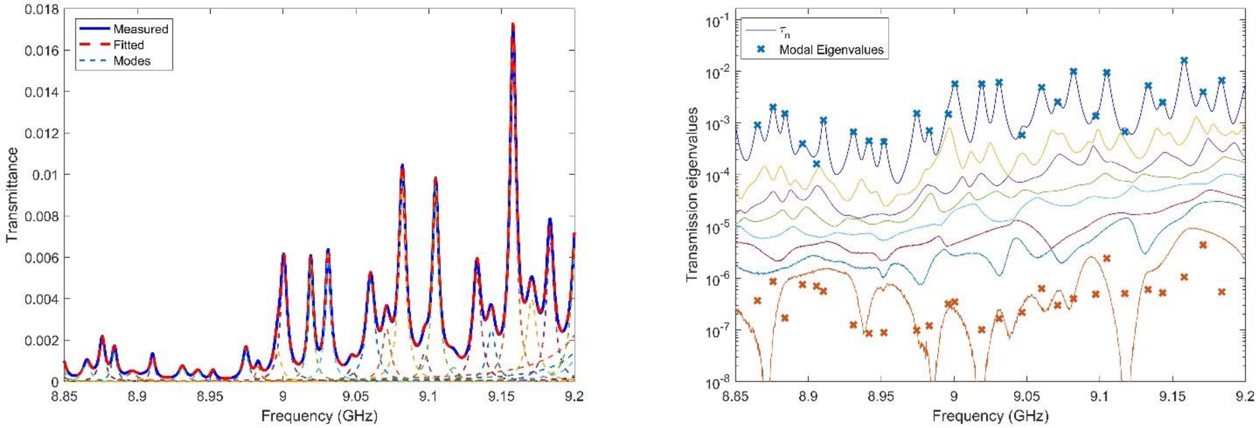

Supplementary Figure 2| Decomposition of measured spectra into MTMs. (a) Transmittance (blue line) and its reconstruction found using HI (dashed red line) found from measurement of the transmission matrix in a weakly scattering sample between 8.5 and 10 GHz (b) The corresponding transmission eigenvalues and the two first eigenvalues of the MTM are shown in a semilog scale.

Supplementary Note 2 – Coupling between eigenfunctions in finite-element simulations.

To further explore the impact of the mixing of wavefunctions of the closed system upon the degree of modal selectivity for an open disordered system, we carry out two-dimensional simulations in COMSOL to solve Maxwell's equations and compute i) the eigenfrequencies and the associated field patterns and ii) the spectrum of the transmission matrix $t(\omega)$. The scattering disks are included in a waveguide with perfectly reflecting side walls. The waveguide supports $N = 9$ channels (see Fig. 5). Two different configurations are considered. The two samples are each a collection of 220 disks of radius $r = 0.18\lambda_0$ randomly positioned inside a waveguide of width $W = 4.5\lambda_0$. The wavelength λ_0 is at the frequency $f_0 =$ 10 GHz. The sample lengths and relative permittivities are $L = 35\lambda_0$ and $\epsilon_r = 2.3$ for the first sample and $L = 25\lambda_0$ and $\epsilon_r = 4.8$ for the second sample.

The eigenfrequencies are found using the Eigenfrequency solver. Open boundary conditions are simulated using Perfectly Matched Layers (PML) at the left and right sides of the waveguide. The TM is then simulated using the frequency domain solver over the range 9.7-10 GHz. Boundary conditions are transverse electric rectangular ports.

For the first sample, we first consider a case of two modes with modal overlap $\delta_n = 0.55$. The square of the real and imaginary parts of the field patterns ϕ_1 and ϕ_2 found in first step normalized so that $\langle \phi_n^* | \phi_n \rangle = 1$, are shown in Fig. SM3 and are seen to extend throughout the sample. Here, $\langle \mathrm{Im}(\phi_n)^2 \rangle \ll \langle \mathrm{Re}(\phi_n)^2 \rangle$, so that the degree of complexness is small for both modes, $q_1^2 = 0.07$ and $q_2^2 = 0.03$. The two eigenfunctions are seen to be very different. Transmission spectra corresponding to maximal coupling for each of the two modes are shown in Fig. SM3(b,d). Transmission is close to unity at resonance with the chosen mode while the contribution of the other mode is small. Because of the small correlation between the modes, it is possible to discriminate between the two modes.

However, when two modes overlap spectrally, the ability to exclusively select one of the modes is reduced. Two hybridized modes, which are double-peaked inside the sample, are found using the second sample and are shown in Fig. SM3(e,g). The degree of spectral overlap between the modes is $\delta_n = 1.13$. $\mathrm{Re}(\phi_1)$ and $\mathrm{Im}(\phi_2)$, and $\mathrm{Im}(\phi_1)$ and $\mathrm{Re}(\phi_2)$, are similar as expected from Eq. (10) of the main text for $f \neq 0$. The eigenfunctions give $q_1^2 = 0.17$ and $q_2^2 = 0.16$. The two values are close but not precisely equal because of the influence of other modes that overlap weakly. The extended modes in the localized regime are 'necklace states' which exhibit multiple peaks within the sample due to the hybridization of spectrally overlapping localized states [1-5]. Such states with high transmission and broad linewidth are rare but contribute substantially to average transmission. We observe in Figs. SM3(f,h) that the transmission spectra and the contribution of the two modes maximally coupled to the first or second mode are nearly the same so that it is not possible to discriminate the two modes . In this case, the correlation between the incident waveforms W_{L1} and W_{L2}, $\left|W_{L1}^{\dagger} W_{L2}\right| / (\|W_{L1}\| \|W_{L2}\|)$ is 0.92.

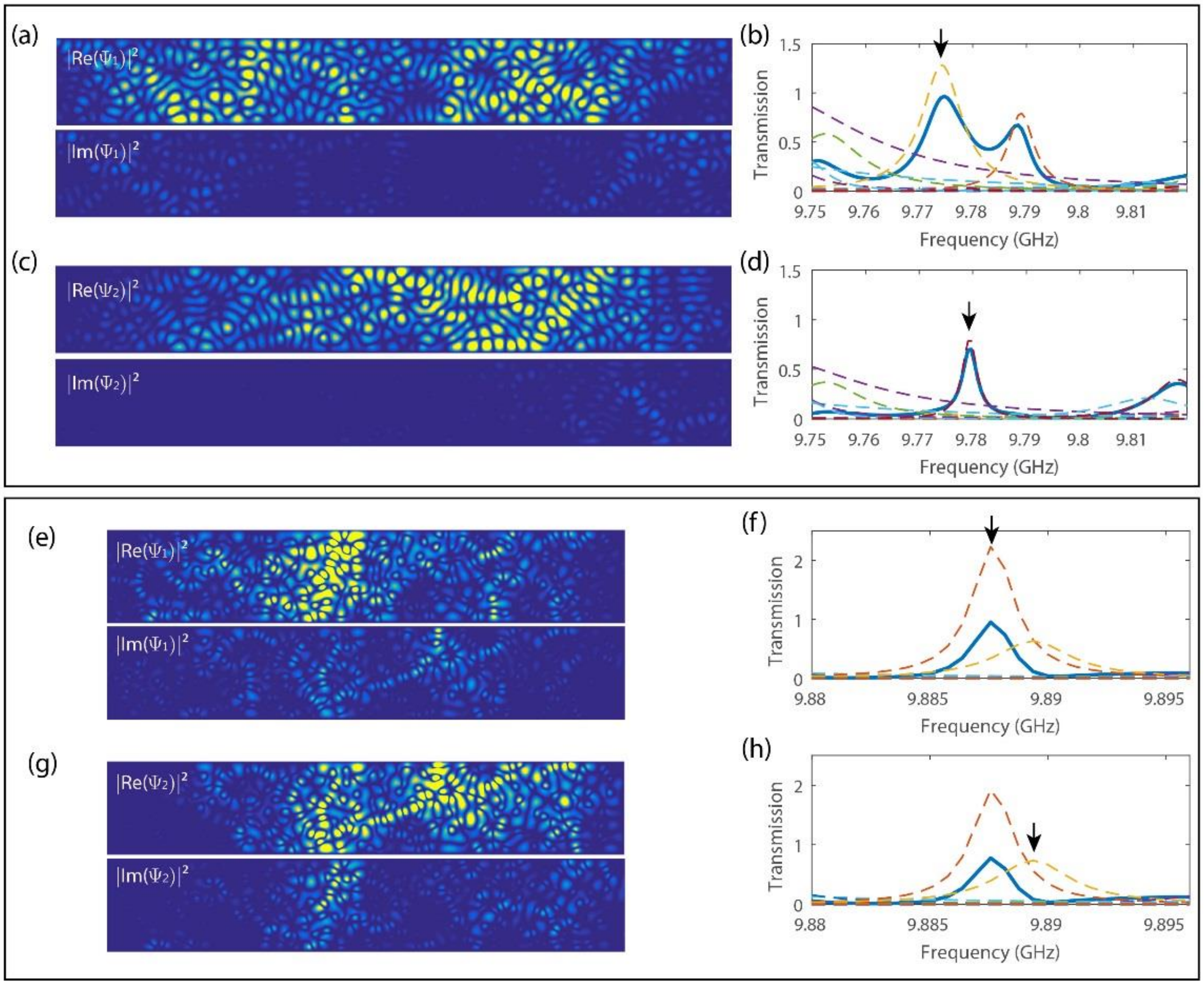

Supplementary Figure 3| Simulation of the modal mixing in disordered media. (a-d) Two modes of the first sample. (a,c) square of the real and imaginary parts, $\mathrm{Re}(\phi_n)^2$ and $\mathrm{Im}(\phi_n)^2$, of two modes with resonance at $f_1 = 9.774$ GHz and f_2 =9.779 GHz with linewidths $\Gamma_1/(2\pi) = 4.91$ MHz and $\Gamma_2/(2\pi) = 1.73$ MHz, respectively. (b,c) Transmission spectra for maximal coupling to the first (b) and the second (d) modes. The dashed lines are the modal strengths. (e-h) Two modes of the second sample: same as (a-d) but for two modes at $f_1 = 9.887$ GHz and $f_2 = 9.889$ GHz with linewidths $\Gamma_1/(2\pi) = 0.84$ MHz and $\Gamma_2/(2\pi) = 2.32$ MHz exhibiting strong modal mixing.

Supplementary Note 3 - Degree of modal control using the two-level effective Hamiltonian.

We derive the parameter η giving the strength of the neighboring mode relative to the strength of the mode to which the incident wavefront is maximally coupled at the resonance. This requires an estimate of the parameters $\left|\mathbf{W}_{\mathrm{L1}}^{\dagger}\mathbf{W}_{\mathrm{L1}}\right|^2$ and $\left|\mathbf{W}_{\mathrm{L2}}^{\dagger}\mathbf{W}_{\mathrm{L1}}\right|^2$ involved in Eq. (11) of the main text. To this end, using the decomposition of the eigenvectors $|\boldsymbol{\phi}_n\rangle$ of $\mathbf{H}_{\mathrm{eff}}$ in the basis $\{|\boldsymbol{\psi}_n\rangle\}$ of the unperturbed eigenvectors of the Hamiltonian of the closed system $\mathbf{H}_0$, we approximate the coupling vectors $\mathbf{W}_{\mathrm{L1}}$ and $\mathbf{W}_{\mathrm{L2}}$

$$\mathbf{W}_{L1} \sim \frac{\mathbf{W}_{L1}^{0} - if\mathbf{W}_{L2}^{0}}{1-f^2}\ ,\ \mathbf{W}_{L2} \sim \frac{\mathbf{W}_{L2}^{0} + if\mathbf{W}_{L1}^{0}}{1-f^2} \tag{1}$$

The vectors W_{Ln}^{0} correspond to the projection of the modes of the closed system onto the left leads of the open system. For $N \gg 1$, we assume that those vectors are statistically independent so that the scalar product between vectors with indexes n and k is, $\mathbf{W}_{n}^{0\dagger}\mathbf{W}_{k}^{0} \sim \sigma^2\delta(n-k)$. Using Supplementary Eq (1), in the limit $f \ll 1$, gives $\mathbf{W}_{L1}^{\dagger}\mathbf{W}_{L1} \sim \sigma^2$ and $\mathbf{W}_{L2}^{\dagger}\mathbf{W}_{L1} \sim -2if\sigma^2$ to first order, and, $\left|\mathbf{W}_{L2}^{\dagger}\mathbf{W}_{L1}\right|^2 / \left|\mathbf{W}_{L1}^{\dagger}\mathbf{W}_{L1}\right|^2 \sim 4f^2$. Finally this gives

$$S_{opt} \sim \frac{1}{1+\frac{|\varphi_1(\omega_1)|^2}{|\varphi_2(\omega_1)|^2}4f^2} \tag{2}$$

Using that $\varphi_n(\omega) = \left(\omega - \omega_n + \frac{i\Gamma_n}{2}\right)^{-1}$ leads to the result $S_{opt} \sim \left[1 + \frac{4f^2\Gamma_1^2}{4\Delta_{12}^2+\Gamma_2^2}\right]^{-1}$ given in the main text, where $\Delta_{12} = \omega_2 - \omega_1$. Supplementary Eq. (2) shows that S_{opt} is the product of a term giving the modal overlap between the two modes and a second term giving the mode mixing.

Supplementary References

[1] K. Y. Bliokh, Y. P. Bliokh, V. Freilikher, A. Z. Genack and P. Sebbah, Coupling and Level Repulsion in the Localized Regime: From Isolated to Quasiextended Modes, *Phys. Rev. Lett.* **101,** 133901 (2008).

[2] C. Vanneste and P. Sebbah, Complexity of two-dimensional quasimodes at the transition from weak scattering to Anderson localization, *Phys. Rev. A* **79,** 041802 (2009).

[3] P. Sebbah, B. Hu, J. M. Klosner and A. Z. Genack, Extended Quasimodes within Nominally Localized Random Waveguides, *Phys. Rev. Lett.* **96,** 183902-183904 (2006).

[4] J. Pendry, Quasi-extended electron states in strongly disordered systems, *J. Phys. C* **20,** 733 (1987).

[5] J. Bertolotti, S. Gottardo, D. S. Wiersma, M. Ghulinyan and L. Pavesi, Optical Necklace States in Anderson Localized 1D Systems, *Phys. Rev. Lett.* **94,** 113903 (2005).
